# Phylogenetic Structure of Tree Species across Different Life Stages from Seedlings to Canopy Trees in a Subtropical Evergreen Broad-Leaved Forest

**DOI:** 10.1371/journal.pone.0131162

**Published:** 2015-06-22

**Authors:** Yi Jin, Hong Qian, Mingjian Yu

**Affiliations:** 1 College of Life Sciences, Zhejiang University, Hangzhou, Zhejiang, China; 2 Research and Collections Center, Illinois State Museum, Springfield, IL, United States of America; Wuhan Botanical Garden,CAS, CHINA

## Abstract

Investigating patterns of phylogenetic structure across different life stages of tree species in forests is crucial to understanding forest community assembly, and investigating forest gap influence on the phylogenetic structure of forest regeneration is necessary for understanding forest community assembly. Here, we examine the phylogenetic structure of tree species across life stages from seedlings to canopy trees, as well as forest gap influence on the phylogenetic structure of forest regeneration in a forest of the subtropical region in China. We investigate changes in phylogenetic relatedness (measured as NRI) of tree species from seedlings, saplings, treelets to canopy trees; we compare the phylogenetic turnover (measured as β_NRI_) between canopy trees and seedlings in forest understory with that between canopy trees and seedlings in forest gaps. We found that phylogenetic relatedness generally increases from seedlings through saplings and treelets up to canopy trees, and that phylogenetic relatedness does not differ between seedlings in forest understory and those in forest gaps, but phylogenetic turnover between canopy trees and seedlings in forest understory is lower than that between canopy trees and seedlings in forest gaps. We conclude that tree species tend to be more closely related from seedling to canopy layers, and that forest gaps alter the seedling phylogenetic turnover of the studied forest. It is likely that the increasing trend of phylogenetic clustering as tree stem size increases observed in this subtropical forest is primarily driven by abiotic filtering processes, which select a set of closely related evergreen broad-leaved tree species whose regeneration has adapted to the closed canopy environments of the subtropical forest developed under the regional monsoon climate.

## Introduction

Understanding how the phylogenetic structure of a forest changes as individual plant development proceeds within the forest and what role forest gaps play in driving phylogenetic community structure of forest regeneration is crucial to understanding the assembly processes that shape forest structure and species composition [1–3]. Within a forest, the formation of local assemblage might be affected by multiple assembly processes, including abiotic filtering, biotic interactions and neutral processes [1, 4–6]. Among the species of a regional species pool that could disperse into a local assemblage, abiotic filtering tends to select ecologically similar species [7–8]; biotic interactions might exclude both ecologically similar species [9–10] and ecologically dissimilar species [11–13]; and neutral processes disregard ecological differences among species [5, 14]. Depending on the degree of phylogenetic conservatism of critical ecological traits, species ecological similarities can be approximated by species phylogenetic relatedness [15–16]. Specifically, a clustered phylogenetic structure could be observed when abiotic filtering is a dominant force in community assembly, causing species to be more closely related than expected by chance [1, 15], although competitive exclusion driven by fitness difference may also lead to clustered phylogenetic structure [11]; an overdispersed phylogenetic structure (i.e., co-occurring species are more distantly related than expected by chance) could be caused by niche partitioning driven by biotic interactions [11, 15], facilitation or abiotic filtering on convergently evolved clades [1]; a random phylogenetic structure can result from neutral processes [5], lack of phylogenetic conservatism, or combined effects of abiotic filtering and biotic interactions [17].

Different size classes of tree stems within a primary (or old-growth) forest stand may be considered as a putative proxy for forest temporal dynamics [3, 18–19]; thus investigating changes in phylogenetic community structure across tree size classes might shed light on the community assembly processes during individual plant development [18–20]. Specifically, an increasingly overdispersed phylogenetic structure as tree stem size increases might be due to differential mortality caused by competition (e.g., [20–22]); an increasingly clustered phylogenetic structure might be due to differential mortality driven by abiotic filtering (e.g., [15–16, 23]), or could be due to differential mortality caused by fitness difference [11]. However, nearly all relevant previous studies were conducted in tropical regions, where abiotic filtering may not play an important role in governing community assembly [24]. Furthermore, few studies have included seedlings (but see [19, 25]). Because tree seedlings represent the initial developmental stage of a forest, including tree seedlings in a study investigating changes in phylogenetic structure through individual plant development would increase our understanding of community assembly and dynamics.

The subtropical zone, which is a transitional zone between tropical and temperate zones, is narrow in all regions of the world except in China where it covers a large area (~2.5 million km^2^) due to the monsoon climate primarily caused by the uplift of the Qinghai-Tibetan Plateau [26]. This region is characterized by highly complex topography [26], and topographic complexity has been detected to be positively related to species niche differentiation [27]. We expect abiotic filtering in this region to be stronger than the tropics in general. Biotic interactions in the tropics are expected to be stronger than the extratropics [28]. Therefore, the relative strength of abiotic filtering might be stronger and biotic interactions might be weaker in subtropical regions than the tropics. As a result, the increasingly overdispersed phylogenetic structure as tree stem size increases, which has been frequently observed in forests of the tropics (e.g., [18, 20]), might switch to increasingly clustered phylogenetic structure as tree stem size increases in forests from the subtropical region (expectation 1). A previous study on subtropical forests has indeed shown that phylogenetic relatedness of tree species increases from saplings to canopy trees [29], although that study did not include seedlings.

Previous studies have shown that environment is a major determinant of phylogenetic structure of communities [20, 30–32]. Forest gaps are commonly formed in primary (or old-growth) forests due to canopy tree fall. Forest gap environments are characterized by higher resource (e.g., light) levels and lack of canopy protection from harsh weather conditions as compared with forest understory [33], which resembles to some degree the early successional forest habitats [34]. Theoretically, early successional habitats are expected to favor pioneer species that are adapted to a high resource environment, while species interactions are expected to be relatively weak [35–38]. As a result, one would expect to find higher phylogenetic relatedness among species within communities in forest gaps than in forest understory (expectation 2). Further, previous studies have shown that phylogenetic structure of communities is scale-dependent [18]. Environmental conditions in forest gaps are positively related to forest gap size [39], thus large forest gaps would resemble to a larger degree the early successional forest habitats than small forest gaps [34], one would expect to find increasing phylogenetic relatedness among species within communities in forest gap as the size of forest gap increases (expectation 3). In addition, it is expected that canopy trees in primary (or old-growth) forests mainly are primary species that are adapted to regenerate in forest understory [40]. Therefore, if forest understory favors primary species regeneration while forest gap favors pioneer species regeneration, seedlings in forest gap might be less similar to canopy trees, compared with seedlings in forest understory. We would expect to find higher phylogenetic turnover between canopy trees and forest gap seedlings than between canopy trees and forest understory seedlings (expectation 4).

In this study, corresponding to the four expectations noted above, we intend to test the following four predictions for a forest in subtropical China: (1) Phylogenetic relatedness of tree species increases (rather than decreases as observed in [18, 20] in tropical forests) with increasing size classes of tree stems in the subtropical forest of this study. (2) Phylogenetic relatedness of seedlings in a forest gap is higher than that under the canopy of the forest surrounding the forest gap. (3) The size of a forest gap is positively related to the phylogenetic relatedness of seedlings in the forest gap. (4) Phylogenetic turnover (i.e., phylogenetic beta diversity) between seedlings under the canopy of a forest and trees in the canopy of the forest is smaller than phylogenetic turnover between seedlings in a forest gap within the forest and trees in the canopy of the forest.

## Materials and Methods

### The study area and sampling plots

Our study site was located at the Gutianshan National Nature Reserve (29°10'19"–29°17'41" N, 118°03'50"–118°11'12" E) in Zhejiang Province, China. The reserve is ~81 km^2^ in area, and was set up to protect the typical old-growth evergreen broad-leaved forest (EBLF) in this humid subtropical region. Local annual mean temperature is 15.3°C, with January minimum temperature being −6.8°C and July maximum temperature being 38.1°C; annual mean precipitation recorded from 1958 to 1986 was 1964 mm [41]. In 2005, a 24-ha (400 m × 600 m) forest dynamics plot (FDP; 29°15'6"–29°15'21" N, 118°7'1"–118°7'24" E; [42]) of evergreen broad-leaved forest was set up in the core area of the reserve following the protocol of the Center for Tropical Forest Science [43]. The forest is dominated by such evergreen broad-leaved tree species as *Castanopsis eyrei* (maximum diameter at breast height of 87.4 cm, relative abundance 8.81%) and *Schima superba* (83.0 cm, 6.04%) and is a typical EBLF. All individuals of tree species with diameter at breast height (DBH) ≥ 1 cm were identified, measured, and georeferenced during the summer of 2005. Detailed descriptions for the reserve and FDP can be found in Zhu et al. [42]. The Administration Bureau of Gutianshan National Nature Reserve issued the permission of carrying out this study.

The FDP experienced a severe ice-storm damage in 2008 [44], leading to numerous canopy openings (i.e., forest gaps). During the summer of 2010, we surveyed the structure and distribution of forest gaps caused by the ice storm within the FDP. We determined forest gaps following Brokaw’s [45] definition of forest gap (i.e., a vertical hole extending from the canopy down to an average height of about two meters above ground). The area of a forest gap was estimated using Runkle’s [46] approximation method. Because the vast majority (84.3%) of the FDP was located in two topographic habitat types (i.e., low valleys and low ridges), we confined our sampling within these two topographic habitats. We divided the identified gaps into four size classes: 25–100 m^2^, 100–200 m^2^, 200–300 m^2^, and 300–500 m^2^ [47–48]. Within each size class of each topographic habitat type, we randomly selected four gaps, which resulted in a total of 32 forest gaps.

For each forest gap, we set up a sampling plot which included all area within the radius of 30 m from the center of the forest gap. This 30-m distance threshold was chosen to avoid density-dependent effects within a distance of 20 m [49] on one hand and not to be too large so that including different micro-habitat types within a sampling plot was avoided or minimized on the other hand. We obtained the data of tree species composition and abundance for all individuals with DBH ≥ 1 cm from the database resulting from the 2005 survey (see above). Within each sampling plot, we set up numerous sampling quadrats of 1 × 1 m for investigating seedlings; 905 sampling quadrats were set up for the 32 sampling plots. Within each sampling plot, sampling quadrats were separated by 2.5−5 m in distance and were evenly distributed across both forest understory and forest gap. The number of sampling quadrats varies among sampling plots (28.28 ± 6.91 SD). We tagged and identified every alive seedling (< 1 cm DBH) of tree species within the quadrats in August of 2011, and reinvestigated the seedlings in August of 2013. In this study, we included those seedlings that were alive at the times of both 2011 and 2013 surveys to assure that all seedlings used in this study were established at least for two years. We divided all recorded individuals of trees in a sampling plot into four stem size classes: seedlings (DBH < 1 cm), saplings (1 cm ≤ DBH < 5 cm), treelets (5 cm ≤ DBH < 10 cm); trees (DBH ≥ 10 cm; for ease of discussion, hereafter, we called them “canopy” trees although some of them may not enter forest canopies). For each sampling plot, sampling quadrats under the forest canopy of the plot were combined to generate a list of species with the number of seedlings per species documented; similarly, sampling quadrats in the forest gap were also combined to generate a list of species with the number of seedlings per species documented; seedlings under the forest canopy were compared with those in the forest gap.

One hundred and twenty one (85.2%) of the 142 angiosperm tree species in the entirety of the 24-ha Gutianshan FDP were found in the 32 sampling plots, although the sampling plots covered only less than 37.7% of the total area of the FDP. The relative abundance of tree species in major clades (e.g., Fagales, Laurales, and Ericales) in the 32 sampling plots was consistent with that in the entire FDP not only for the combination of all tree stems of sapling, treelets, and canopy trees but also for each of the three groups of tree stems (τ = 1, *P* < 0.05 in all cases, Kendall’s τ test for correlations; see S1 Fig).

### Species pool and phylogenetic tree

The 24-ha Gutianshan FDP comprises 144 tree species, 142 of which are angiosperms. These species were described as either trees or small trees in *Flora of China* (http://www.efloras.org/) and Zheng [50] and are larger than 10 cm DBH at their maturity. We followed many previous studies (e.g., [3, 51]) to exclude non-angiosperm species because non-angiosperm species are few in each sampling plot (only two gymnosperm species in the Gutianshan FDP) but their much longer branches in a phylogeny, compared to those of angiosperms, would contribute to unusually high phylogenetic measures [3] and thus may obscure phylogenetic patterns of angiosperms. The 142 angiosperm tree species were included in the species pool of all phylogenetic analyses in this study. These species belong to 89 genera in 40 families and 21 orders [52].

The phylogeny used in this study was pruned from the phylogeny constructed for the Gutianshan FDP based on three common barcoding genes (rbcLa, matK and trnH-psbA; [53]). This phylogeny was well resolved at the species level and included 159 species. Eleven tree species found in our study were absent from the phylogeny; accordingly, we added them to the phylogeny by placing them as polytomies of their most closely related species in the phylogeny (S2 Fig).

### Data analyses

Net relatedness index (NRI) is a commonly used index to quantify phylogenetic relatedness (e.g., [3, 51, 54]). Accordingly, we used NRI in our study. It is defined as [1]:

$$NRI=-1\times \frac{MPD_{sample}-MPD_{random}}{sdMPD_{random}}$$

where MPD is the mean pairwise phylogenetic distance between all species within an assemblage, MPD_sample_ is the observed MPD, MPD_random_ is the expected MPD of randomized assemblages, and sdMPD_random_ is the standard deviation of the MPD for the randomized assemblages. Randomized (null) assemblages were generated by randomly drawing species from the species pool to replace the species in the sample. A positive NRI value indicates that MPD is lower than expected by chance (i.e., species more closely related than expected by chance) and that phylogenetic clustering of species occurs; a negative NRI value results when the observed MPD is greater than expected by chance (i.e., species more distantly related than expected by chance) and thus indicates phylogenetic evenness or overdispersion. For each NRI value, 999 randomized assemblages were generated. Species were weighted by their abundance (i.e., the number of individuals) within each assemblage. For each sampling plot, we calculated NRI separately for seedlings in the forest gap, seedlings under the forest canopy, saplings, treelets, and canopy trees. In addition to calculating NRI for each assemblage of seedlings with DBH < 1 cm, we also calculated NRI for each assemblage of seedlings with height < 50 cm, which is a stricter definition for seedlings (see [19]). NRI was calculated using the “comstruct” function in the Phylocom 4.2 software [55] and null model 2 in Phylocom 4.2 (i.e., randomly drawing species from the phylogeny pool).

To determine the degree to which phylogenetic turnover between seedlings in the two habitats (i.e., forest understory vs. forest gap) and between those in either habitat and canopy trees, we utilized the β_NRI_ index, which is related to NRI, in analyzing phylogenetic turnover between assemblages [55] with species abundance accounted for. The index represents the negative standardized effect size (−1 × SES) of inter-community mean phylogenetic distance (βMPD). The formula of this index is [55]:

$$\beta_{NRI}=-1\times \frac{\beta MPD_{sample}-\beta MPD_{random}}{sd\beta MPD_{random}}$$

where βMPD is the mean pairwise phylogenetic distance of all species between two assemblages, βMPD_sample_ is the observed βMPD, βMPD_random_ is the expected βMPD between two randomized assemblages, and sdβMPD_random_ is the standard deviation of the βMPD for the two randomized assemblages. For each β_NRI_ value, 999 pairs of randomized assemblages were generated to estimate βMPD_random_ and sdβMPD_random_. Randomized assemblages were generated by randomly sampling species from the species pool to replace the species in the samples (null method 2 in Phylocom 4.2, http://phylodiversity.net/phylocom; [55]). A higher value of β_NRI_ indicates a higher phylogenetic similarity and thus a lower phylogenetic turnover between assemblages compared. β_NRI_ was calculated using the “comdist” function in Phylocom 4.2 [55].

We used Wilocoxon signed-rank test to determine statistic significance for pairwise comparisons [18]. We used Cochran-Armitage approach to test for directional change in the phylogenetic structure with increasing tree stem size, performed by the “independence_test” function of the “coin” package in R software version 3.1.0 [56–57]. We examined whether differences in gap size influenced patterns of phylogenetic structure of seedlings in forest gaps by correlating forest gap size with values of phylogenetic indices for seedlings in forest gap using Kendall’s τ as correlation coefficient [58].

## Results

**Fig 1:**
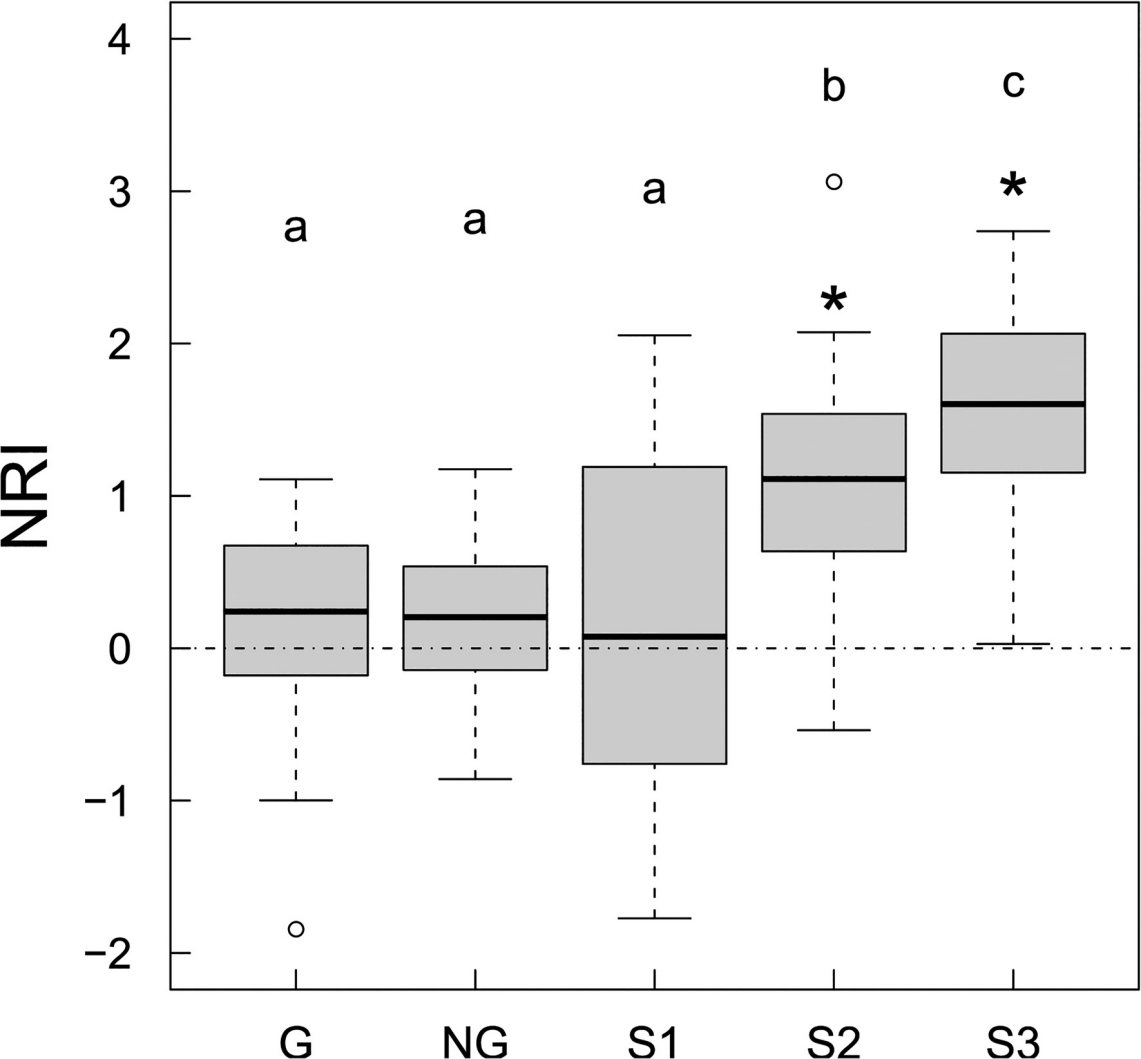
NRI of different size classes of tree stems. Box represents the interquartile range and the median (the thick line); the whiskers extend to the most extreme data point within 1.5 times interquartile range from the box. Values above and below the dotted line indicate phylogenetic clustering and overdispersion, respectively. G stands for seedlings in forest gaps, NG for seedlings in forest understory, S1 for saplings (1 cm ≤ DBH < 5 cm); S2 for treelets (5 cm ≤ DBH < 10 cm), S3 for canopy trees (DBH ≥ 10 cm). Different lower case letters above the boxes indicate significant differences (*P* < 0.05) in multiple comparisons (*P* values were adjusted using the “Bonferroni” method). Asterisks above the boxes indicate that the dispersions significantly differed from zero (*P* < 0.05).

NRI values for seedlings in forest understory and in forest gaps were not significantly different from those expected by chance (Fig 1). NRI values for treelets and canopy trees were significantly higher than expected by chance (Fig 1). NRI values for canopy trees were significantly greater than those for seedlings and saplings (Fig 1). There was a clear trend for NRI to increase with increasing stem size of trees (*P* < 0.001, Cochran-Armitage test for trend for either seedlings in forest gaps, saplings, treelets and trees or seedlings in forest understory, saplings, treelets and trees; Fig 1), indicating an increasing trend in phylogenetic clustering from seedling stratum to forest canopy stratum, regardless of whether seedlings are defined as individuals with DBH < 1 cm or as individuals of < 50 cm in height (compare Fig 1 with S3 Fig). Forest gap size showed no significant influence on patterns of phylogenetic structure of seedlings (*P* > 0.1, Kendall’s τ coefficient for correlations between forest gap sizes and the two phylogenetic measures of seedlings in forest gaps; see S1 Table).

**Fig 2:**
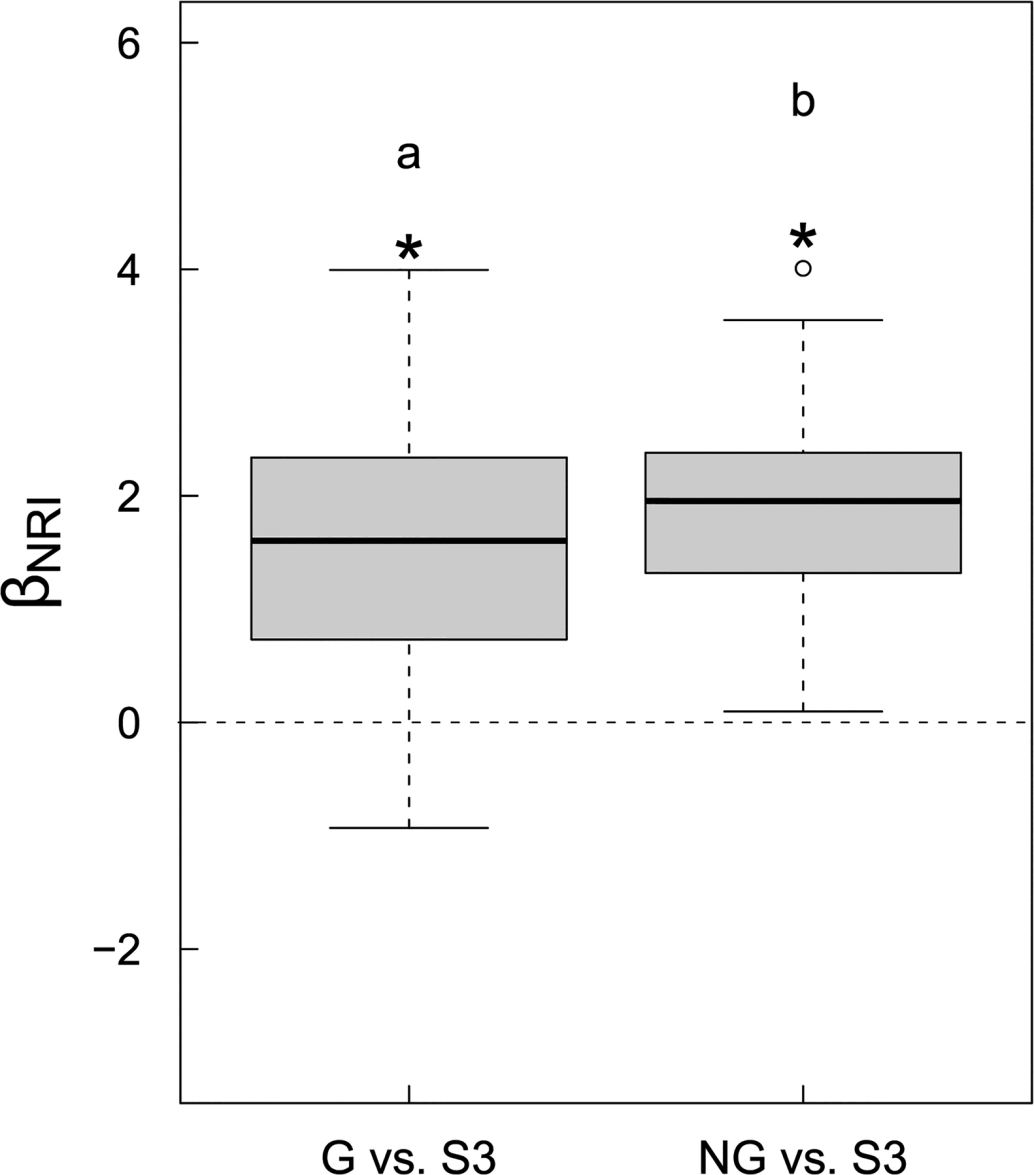
Comparison of β_NRI_ values for paired assemblages of seedlings in forest gaps versus canopy trees (G vs. S3) with those of seedlings in forest understory versus canopy trees (NG vs. S3). Different lower case letters above the boxes indicate significant differences (*P* < 0.05).

The mean value of β_NRI_ between seedlings and canopy trees was significantly greater than zero regardless of whether seedlings under forest canopies or in forest gaps were considered (Fig 2). Values of β_NRI_ between seedlings in forest gap and canopy trees were significantly lower than those between seedlings in forest understory and canopy trees (*P* < 0.05, Wilocoxon signed-rank test; Fig 2). In other words, the analysis using β_NRI_ indicates that phylogenetic turnover was higher between seedlings in forest gap and canopy trees than between seedlings in forest understory and canopy trees.

## Discussion

Since an early attempt of performing a phylogenetic analysis of community structure [15], the use of phylogenetic approaches in investigating patterns and processes of community assembly has blossomed [59]. Investigating patterns of phylogenetic structure among different stem size classes of tree species within forests can gain insights into mechanisms of forest community assembly. Several studies (e.g., [18–20]) have investigated phylogenetic patterns of size classes of tree species, but nearly all of the studies are restricted to wet tropical regions where climatic conditions are generally benign and abiotic filtering is generally a minor factor driving community assembly of forests, compared to forests outside of tropical regions [24, 60]. Furthermore, seedlings of tree species are important to the formation and development of a forest, but to our knowledge only two of the studies that investigated patterns of phylogenetic structure across a series of different size classes of tree stems have included seedlings [19, 25]. To our knowledge, our study is the first to investigate patterns of phylogenetic structure across different size classes from seedlings to canopy trees in old-growth forests of subtropical regions. In addition, we have investigated the phylogenetic structure of seedlings both in forest understory and in forest gaps.

In the subtropical forest of our study, we found increasingly clustered phylogenetic structure as stem size increases. Significant phylogenetic clustering patterns were observed even in the treelet size class, a size class prior to the canopy tree size class (Fig 1). Our finding is consistent with Webb et al.’s [19] finding for a tropical forest, where they found increasingly clustered phylogenetic structures with increasing plant developmental stages from seedlings to adult trees. Our finding is also in agreement with that of Feng et al. [29] for angiosperm trees in undisturbed forests in the same subtropical region as our study, although their study did not include seedlings and differences in NRI among the three stem size classes in their study are statistically not significant.

Although 44% of the 142 angiosperm tree species included in our study site (i.e., the 24-ha Gutianshan FDP) are deciduous species, nearly all canopy trees are evergreen broad-leaved species. The relative abundance of trees with DBH ≥ 10 cm in our study site was 78.1% for evergreen broad-leaved trees, 15.4% for deciduous broad-leaved trees, and 6.5% for coniferous trees. These characterize a typical feature of the primary (or zonal) EBLFs in this region. Many of the canopy tree species are congeners, confamilials, or taxa in closely related families. For example, of the 80 evergreen broad-leaved tree species found in our study site, ten species are in Lauraceae and nine species are in Fagaceae, eight of which belong to *Castanopsis*, *Cyclobalanopsis* and *Lithocarpus* which are typical dominant canopy trees in Chinese subtropical forests, and *Castanopsis eyrei* is the most dominant canopy tree species in our study site. In addition, nine species in Theaceae found in our study site are evergreen. Evergreen broad-leaved tree species of these families and other typical subtropical-distributed families such as Aquifoliaceae (with ten evergreen species in our study site) are the dominant species forming the canopies of forests in this subtropical region [26]. Our study has shown clear shifts in the relative abundance of tree species in some major clades. For example, species in the order Fagales (especially in the family Fagaceae) are the most common taxa of forest canopies in our study site; the relative abundance of the species in Fagales clearly increases along the series from saplings to canopy trees (S1 Fig). Conversely, the relative abundance of the species in the order Ericales decreases along the same series (S1 Fig).

Previous studies have shown that phylogenetic structures of early successional forest communities are often more clustered than primary or old-growth forest communities in tropical regions [3, 21, 54, 61] as well as in the subtropical region [29] where our study was conducted. In the old-growth forest, because forest gaps are disturbed habitats and microenvironmental conditions (e.g., light, air temperature, soil moisture) in forest gaps may resemble to some degree the initial stage of forest succession [34], one would expect to find a higher degree of phylogenetic relatedness for seedlings in forest gaps than seedlings in forest understory [22, 62]. However, we did not find support for this prediction in the forest of our study. Further, because resource levels (e.g., light) in forest gaps are positively related to gap size [39], large gaps might resemble the early successional forest environments to a larger degree than small gaps and thus gap size might be positively related to seedling phylogenetic relatedness. However, we did not find support for this prediction either. Du et al. [63] compared the seed arrival patterns in forest gaps with forest understory in the studied forest, and found that both forest gaps and forest understory were subjected to dispersal limitation. We suspect that the lack of influence of forest gap as compared with forest understory and the lack of influence of forest gap size on seedling phylogenetic relatedness in this forest might be partly due to the prevalent limitation in seed dispersal in both forest gaps and forest understory of this forest [63]. Furthermore, some of the seedlings in forest gaps might be the advance regeneration that recruited before the formation of forest gaps, which would to some extent mask the effects of forest gap environments on seedling recruitment. Nonetheless, we found that phylogenetic turnover between canopy trees and seedlings in forest understory was slightly lower than that between canopy trees and seedlings in forest gaps (Fig 2), which suggests that forest gap favored the establishment of species that are phylogenetically more dissimilar with canopy trees than forest understory.

In sum, our study shows that phylogenetic relatedness generally increases from seedlings through saplings and treelets up to canopy trees in the humid subtropical forest that we investigated. Our study also shows that phylogenetic relatedness does not differ between seedlings in forest understory and those in forest gaps, but phylogenetic turnover between canopy trees and seedlings in forest understory is lower than that between canopy trees and seedlings in forest gaps. Evergreen broad-leaved forests of subtropical regions are an important type of forests not only in China [64] but also in the world [65]. Because our study is, to our knowledge, the first to investigate phylogenetic structure for humid subtropical evergreen broad-leaved forests that includes all vegetation strata from seedlings to canopy trees, we believe our study would help to gain a fuller understanding of mechanisms of community assembly of forests in extratropical regions.

## Supporting Information

S1 Fig Relative abundance of five major orders in three stem size classes for the entire 24-ha Gutianshan FDP (A) and the 32 sampling plots (B). (DOC)

S2 Fig The phylogenetic tree of the 142 angiosperm tree species found in the 24-ha Gutianshan FDP. The following species in the phylogeny are additional to Liu et al.’s (2013) phylogeny: *Castanea henryi*, *Cerasus discoidea*, *Euonymus centidens*, *Fraxinus chinensis*, *Houpoea officinalis*, *Ilex formosana*, *Ilex litseifolia*, *Maclura tricuspidata*, *Mallotus apelta*, *Meliosma rigida*, and *Trema cannabina* var. *dielsiana*.
(DOC)

S3 Fig NRI of different size classes of tree stems. G stands for seedlings of < 50 cm tall in forest gaps, NG for seedlings of < 50 cm tall in forest understory. See Fig 1 for detailed interpretations.
(DOC)

S1 Table Correlations between forest gap sizes and phylogenetic measures of seedlings in forest gaps. (DOC)

S2 Table Data of NRI and βNRI. ψ, seedling is defined as individual with DBH < 1 cm; φ, seedling is defined as individual with height < 50 cm. See Fig 1 and Fig 2 for key to abbreviations.
(XLS)

S3 Table Abundances of the five major clades in the entire FDP and the 32 sampling plots. See Fig 1 for key to abbreviations.
(XLS)
