# Supplementary material for: Phylogenetic Structure of Tree Species across Different Life Stages from Seedlings to Canopy Trees in a Subtropical Evergreen Broad-Leaved Forest
Source: PLoS One. 2015 Jun 22;10(6):e0131162. doi: 10.1371/journal.pone.0131162 (PMC4476806; doi:10.1371/journal.pone.0131162)
Supplement: S1 Fig — (DOC) [file pone.0131162.s001.doc]

**S1 Fig. Relative abundance of five major orders in three stem size classes for the entire 24-ha Gutianshan FDP (A) and the 32 sampling plots (B).**
