# Supplementary figures and images for: Phylogenetic Structure of Tree Species across Different Life Stages from Seedlings to Canopy Trees in a Subtropical Evergreen Broad-Leaved Forest
Source: PLoS One. 2015 Jun 22;10(6):e0131162. doi: 10.1371/journal.pone.0131162 (PMC4476806; doi:10.1371/journal.pone.0131162)

**S2 Fig. The phylogenetic tree of the 142 angiosperm tree species found in the 24-ha Gutianshan FDP.**


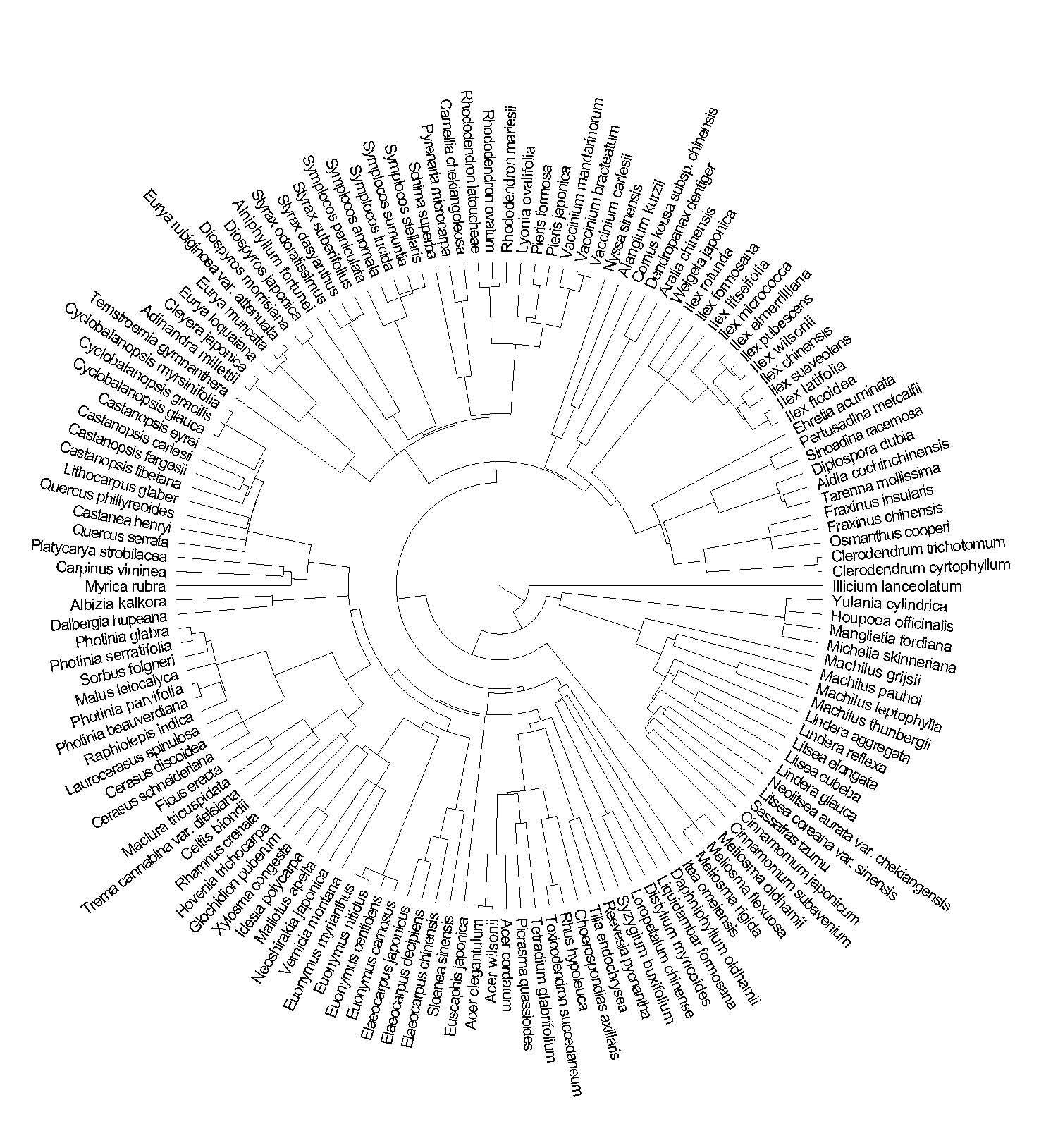

Supplement: S2 Fig — The following species in the phylogeny are additional to Liu et al.’s (2013) phylogeny: Castanea henryi, Cerasus discoidea, Euonymus centidens, Fraxinus chinensis, Houpoea officinalis, Ilex formosana, Ilex litseifolia, Maclura tricuspidata, Mallotus apelta, Meliosma rigida, and Trema cannabina var. dielsiana. (DOC) [file pone.0131162.s002.doc]
