# Supplementary material for: Phylogenetic Structure of Tree Species across Different Life Stages from Seedlings to Canopy Trees in a Subtropical Evergreen Broad-Leaved Forest
Source: PLoS One. 2015 Jun 22;10(6):e0131162. doi: 10.1371/journal.pone.0131162 (PMC4476806; doi:10.1371/journal.pone.0131162)
Supplement: S3 Fig — G stands for seedlings of < 50 cm tall in forest gaps, NG for seedlings of < 50 cm tall in forest understory. See Fig 1 for detailed interpretations. (DOC) [file pone.0131162.s003.doc]

**S3 Fig. NRI of different size classes of tree stems.**
