# Supplementary material for: Phylogenetic Structure of Tree Species across Different Life Stages from Seedlings to Canopy Trees in a Subtropical Evergreen Broad-Leaved Forest
Source: PLoS One. 2015 Jun 22;10(6):e0131162. doi: 10.1371/journal.pone.0131162 (PMC4476806; doi:10.1371/journal.pone.0131162)
Supplement: S1 Table — (DOC) [file pone.0131162.s004.doc]

**S1 Table. Correlations between forest gap sizes and phylogenetic measures of seedlings in forest gaps.**

| Phylogenetic measure | τ | *P* value |
| --- | --- | --- |
| NRI for seedlings in forest gap | −0.065 | 0.618 |
| β_NRI_ between seedlings in forest gap and canopy trees | −0.012 | 0.936 |
